# Right Ventricular Systolic Dysfunction Is Common in Hypertensive Heart Failure: A Prospective Study in Sub-Saharan Africa

**DOI:** 10.1371/journal.pone.0153479

**Published:** 2016-04-13

**Authors:** Dike B. Ojji, Sandrine Lecour, John J. Atherton, Lori A. Blauwet, Jacob Alfa, Karen Sliwa

**Affiliations:** 1 Cardiology Unit, Department of Medicine, University of Abuja Teaching Hospital, Gwagwalada, Abuja, Nigeria; 2 Hatter Institute for Cardiovascular Research in Africa, Department of Medicine, Faculty of Health Sciences, University of Cape Town, Cape Town, South Africa; 3 Department of Cardiology, Royal Brisbane and Women Hospital, and University of Queensland School of Medicine, Brisbane, Australia; 4 Division of Cardiovascular Diseases, Mayo Clinic, Rochester, Minnesota, United States of America; University of Buenos Aires, Cardiovascular Pathophysiology Institute, ARGENTINA

## Abstract

**Introduction:**

Right ventricular (RV) systolic dysfunction is now recognized widely as a strong and independent predictor of adverse outcomes in patients with heart failure (HF). Reduction of RV systolic function more closely predicts impaired exercise tolerance and poor survival than does left ventricular (LV) systolic function. In spite of this, there is a dearth of data on RV function in hypertensive HF which is the commonest form of HF in sub-Saharan Africa. We therefore conducted a prospective cohort study of hypertensive HF patients presenting to the University of Abuja Teaching Hospital, Abuja, Nigeria over an 8 year period.

**Methods:**

Each subject had transthoracic echocardiography performed on them according to the guidelines of American Society of Echocardiography. RV systolic function was defined as a tricuspid annular plane systolic excursion (TAPSE) <15mm using M-mode echocardiography.

**Results:**

RV systolic dysfunction was identified in 272 (44.5%) of the 611 subjects that were studied. Subjects with TAPSE less than 15mm had worse prognosis compared to those with TAPSE ≥15mm.There was a significant correlation between TAPSE and other adverse prognostic markers including left and right atrial area, LV size, LV mass, LV ejection fraction, restrictive mitral inflow and RV systolic pressure (RVSP). However, LV ejection fraction and right atrial area were the only independent determinants of RV systolic dysfunction.

**Conclusions:**

Hypertensive HF is a major cause of RV systolic dysfunction even in a population with a low prevalence of coronary artery disease, and RV systolic dysfunction is associated with poor prognosis in hypertensive HF. Detailed assessment of RV function should therefore be part of the echocardiography evaluation of patients with hypertensive HF.

## Introduction

Right ventricular (RV) systolic dysfunction is now widely recognized as an independent predictor of adverse outcomes in patients with heart failure (HF) especially in those with advanced HF[1]. in whom reduction of RV systolic function more closely predicts impaired exercise tolerance and poor survival than does left ventricular (LV) systolic dysfunction[2,3]. The estimation of RV function is now included in the standard evaluation of patients with HF either due to ischemic heart disease or to primary dilated cardiomyopathy, as this is helpful in the clinical assessment and in the prognostic stratification of such patients[4].

Furthermore, the more widespread use of a simplified and reproducible echocardiography approach to the evaluation of RV function has substantially contributed to the greater awareness of the importance of this chamber [5,6].

In spite of these considerations, RV function in hypertensive HF, which is the commonest form of HF in sub-Saharan Africa [7, 8] has not been well investigated. We therefore undertook a prospective study to determine the prevalence of RV systolic dysfunction in patients diagnosed with hypertensive HF following presentation to the largest hospital in Abuja, Nigeria. We also sought to determine whether RV systolic dysfunction was associated with other adverse prognostic markers.

## Materials and Methods

### Subjects

This prospective cohort study was approved by the University of Abuja Teaching Hospital Ethics Clearance Committee and is in compliance with the Helsinki declaration, besides when feasible adhering to the STROBE guidelines for an observational study of this nature. Written informed consent as approved by the University of Abuja Teaching Hospital Ethical Committee was obtained from all the subjects. The minimum age for participation in the study was 18 years and there was no upper age limit. Recruitment for the present study was initiated in April 2006 and data were obtained until June 2014. The main inclusion criterion was all black hypertensive subjects presenting with acute left-sided HF or biventricular HF for the first time to the emergency department of University of Abuja Teaching Hospital. Subjects with angina symptoms, those with electrocardiography changes of myocardial infarction and those with elevated troponin I were excluded from the study. Subjects with previous acute myocardial infarction, previous diagnosis of heart failure, those with regional wall motion abnormality and valvular lesions on trans thoracic echocardiography, those with diabetes mellitus, and those with serum creatinine levels greater than 170μmol/L were excluded from the study. Hypertension was defined according the JNC VII guidelines [9]. The subjects also had one or more clinical features of long standing hypertension which included thickened arterial wall, locomotor brachialis and at least grade 2 hypertensive retinopathy. HF was diagnosed according to the guidelines of the European Society of Cardiology [10]. The functional status of the HF subjects was categorised according to the New York Heart Association Functional classification[11] Some details of our methodology reproduces information already reported in detail in our previous publication[7].

#### Questionnaire

All the subjects completed a standard questionnaire. Where there was need for language interpretation, both medical and paramedical staff of the Cardiology Unit of the Department of Medicine of the University of Abuja Teaching Hospital assisted. The questionnaire requested specific answers regarding date of birth, gender, occupation, background diagnosis of hypertension, background diagnosis of diabetes mellitus, history of angina, history of alcohol consumption and history of smoking habits. Subjects’ outcome data with respect to length of hospital stay, intra-hospital mortality and one year re-hospitalization were collected prospectively from the subjects’ hospital records and was approved by the University of Abuja Teaching Hospital Ethics Clearance Committee. In keeping with the policy of University of Abuja Teaching Hospital, no informed consent was obtained from these subjects or their next of kin to obtain these records. However, patient records and information was anonymized and de-identified prior to analysis of their data.

#### Anthropometric Measurements and Conventional Blood Pressure Measurement

The height and weight of the subjects were measured during the clinic by Nursing Staff with the participants standing and wearing indoor clothes with no shoes. Body mass index was calculated as weight in kilograms divided by the square of height in meters. Body surface area in meters^2^ (m^2^) was calculated as (0.0001) x (71.84) x (weight in kg) ^0.425^ x (height in cm) ^0.725^

Blood pressure (BP) measurements were obtained according to standard guidelines with a mercury sphygmomanometer (Accouson London). Systolic and diastolic BP were measured by cardiologists at Korotkoff sounds I and V respectively. BP was measured at the right arm three times after the patient has rested in the sitting position for 5minutes, and average of the three measurements was obtained. Pulse pressure was calculated as systolic BP minus diastolic BP while mean arterial pressure was calculated as diastolic BP plus one third of the pulse pressure.

#### Blood Measurements

Blood samples were collected for fasting blood sugar, fasting lipid profile, electrolytes, urea and creatinine, and full blood count.

#### Transthoracic Echocardiography

Subjects had transthoracic echocardiography performed on them using a commercially available ultrasound system (IVIS-60). All the studies were performed by same Cardiologist. Each study was performed with subject in the left lateral decubitus position using standard parasternal, short-axis and apical views according to the recommendations of the American Society of Echocardiography [12]. Left ventricular systolic function was calculated by Teichholz’s formula [13], while left ventricular mass (LVM) was calculated using the formula, LVM = 0.8 [1.04 (IVSTd + EDD + PWT d)^3^ + 0.6g].This formula has been shown[14] to yield values closely related to necropsy LV weight with good inter-study reproducibility (r = 0.90). The diagnosis of LV hypertrophy was said to be present when LVM indexed for height exceeded 49.2g/m^2.7^ in men and 46.7g/m^2.7^ in women [15]. The left and right atria were measured at end-ventricular systole when the atria chambers were at their greatest dimensions, and the RV diameter was measured at diastole using M-mode at the level of the aortic valve. RV systolic function was defined according to the tricuspid annular plane systolic excursion (TAPSE) assessed on echocardiography using M-mode recordings through the lateral tricuspid valve annulus as previously reported [16]. Details of our echocardiography measurements have been previously reported [7].

### Data Analysis

SPSS software version 16.0(SPSS Inc, Chicago, IL) was used for statistical analysis. Continuous variables were expressed as mean± SD. Comparison of demographic, clinical, laboratory and echocardiography parameters among the three groups was performed by analysis of variance. Correlation coefficients were calculated by linear regression analysis with demographic, clinical, laboratory and echocardiography measures being evaluated by Spearman regression. Multivariate linear regression analyses were performed with TAPSE as dependent variable with inclusion of demographic, clinical and echocardiography parameters. A 2-tailed p value < 0.05 was considered significant.

## Results

**Fig 1:**
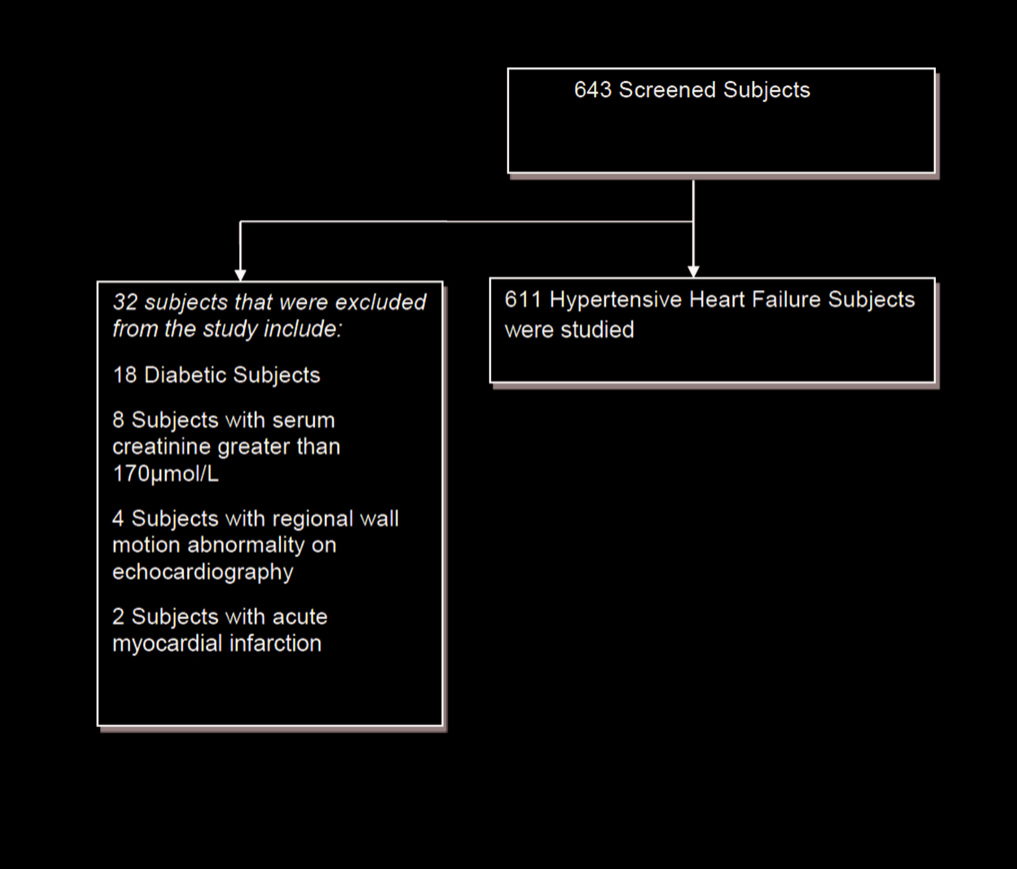
Algorithm of recruitment and enrolment of subjects.

643 patients enrolled for the study but 32 (4.9%) of the total enrolment was excluded because patients were either diabetic, had a regional wall motion abnormality on trans thoracic echocardiography, had serum creatinine levels greater than 170μmol/L or had previous acute myocardial infarction. Therefore, 611 subjects with hypertensive HF were studied. This is shown in Fig 1.

### Clinical Profile of the Subjects

**Fig 2:**
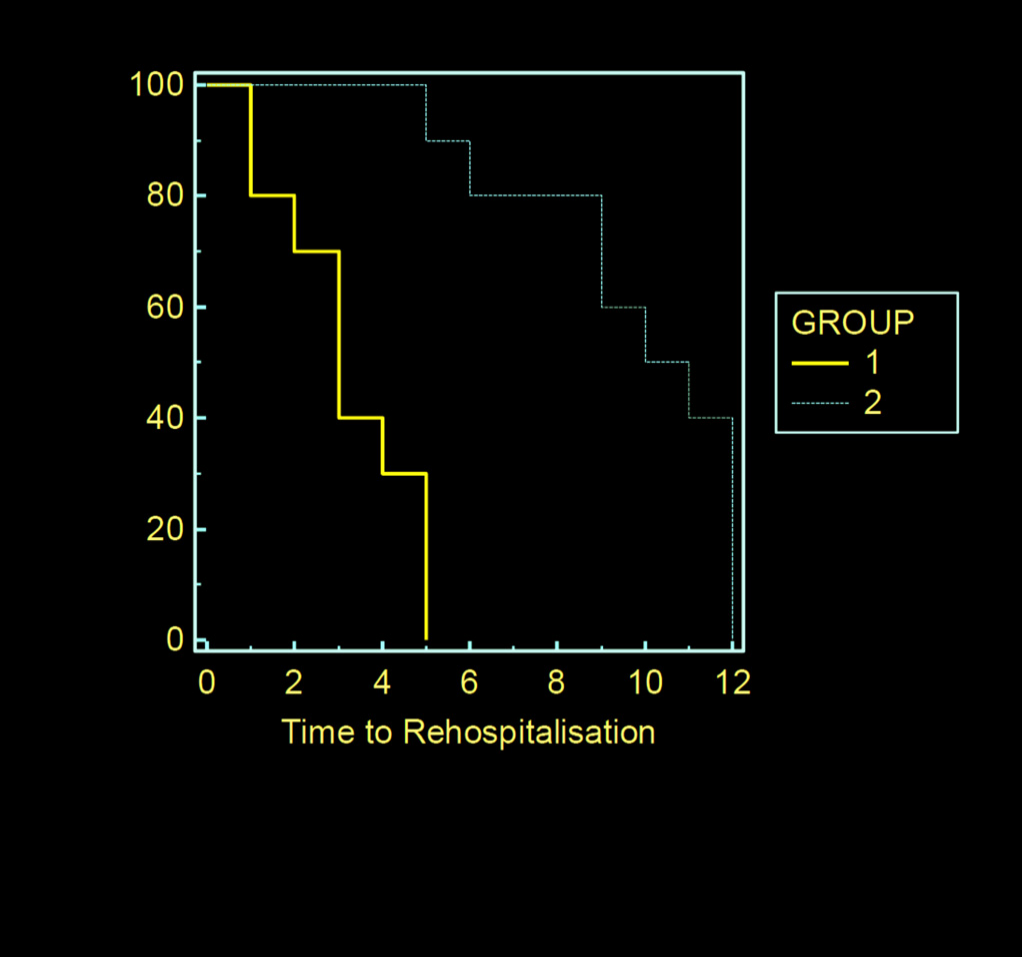
Comparism of the rate of re- hospitalization between subjects with right ventricular systolic function and those with right ventricular systolic dysfunction. Group 1- Subjects with tricuspid annular plane excursion (TAPSE) less than 15mm. Group 2- Subjects with tricuspid annular plane excursion (TAPSE) greater than or equal to 15mm.

Table 1 shows the clinical profile of the subjects studied. The subjects were middle-aged with mean age of 54.8±13.2years. Using TAPSE, 272 (44.5%) of subjects had impaired RV systolic function. Comparing subjects with normal right ventricular (RV) systolic function and those with RV systolic dysfunction, there was no significant difference in age, but subjects with RV systolic dysfunction presented more often with palpitations (57.7% versus 48.9%, p = 0.02) and peripheral oedema, had lower body mass index, lower systolic blood pressure, lower pulse pressure, lower total and LDL cholesterol and worse renal function (estimated glomerular filtration rate 76.5±16.0 versus 111.6 ± 41.4, mL/min, p <0.0001). They were also less likely to be smokers. Subjects with TAPSE < 15mm had longer hospital stay, higher 30-day mortality. They also had higher rate of hospitalization at one year as shown in Kaplan Meir survival curve in Fig 2.

**Table 1:** Clinical Profile of the Subjects.

Parameters	ALL(N = 611)	TAPSE≥ 15mm (N = 339)	TAPSE<15mm (N = 272)	P-value
Age, years	54.8±13.2	55.7±13.4	55.0±13.4	0.57
Smoking Habits, %	24(13.1%)	22(18.6%)	2(2.5%)	<0.001
Female %	268(44%)	158(46.6%)	110 (40.4%)	0.15
Body Mass Index, kg/m^2^	25.30±7.0	26.3±5.4	24.5±6.8	0.0.004
Palpitations, %	204(33.4%)	68(20.0%)	136(50.0%)	0.002
Peripheral Oedema, %	363(59.4%)	136(40.0%)	227(83.3%)	<0.0001
NYHA Class II	111(18.2%)	62(18.5%)	47(17.5%)	
NYHA Class III	389(63.6%)	220(64.8%)	166(60.8%)	
NYHA Class IV	111(18.2%)	57(16.7%)	59(21.7%)	
SBP, mmHg	149.1±23.8	148.1±23.8	142.7±23.6	0.005
DBP, mmHg	98.1±13.9	94.9±16.6	96.4±15.1	0.08
PP, mmHg	55.8±16.2	59.2±18.2	49.3±17.1	<0.0001
MAP, mmHg	101.3±16.4	101.3±17.9	97.9±21.5	0.21
FBS, mmol/L	5.0±0.6	4.9±0.58	4.8±0.56	0.63
Total Cholesterol, mmol/L	4.5±1.2	4.8±.0.8	4.2±1.2	0.013
LDL Cholesterol, mmol/L	3.1±0.9	3.24±0.66	2.7±1.0	0.03
HDL Cholesterol, mmol/L	1.1±0.4	1.2±0.26	1.06±0.3	0.014
Estimated GFR, mls/min/1.73m^2^	101.5±38.8	111.6±41.4	78.3±17.0	<0.0001
Loop Diuretics	568(93.0%)	330(97.3%)	238(87.5)	
Thiazide Diuretics	110(18.0%)	63(18.6%)	47(17.3%)	
Spironolactone	525(85.9%)	285(84.1%)	240(88.2%)	
ACEIS	470(76.9%)	251(74.0%)	219(80.5%)	
ARBS	128(20.9%)	76(22.4%)	52(19.1%)	
Calcium Channel Blockers	165(27.0%)	88(26.0%)	77(28.3%)	
Beta Blockers	86(14.1%)	44(13.0%)	42(15.4%)	
Digoxin	122(20.0%)	59(17.4%)	63(23.2%)	
Warfarin	31(5.1%)	14(4.1%)	17(6.3%)	
Atrial Fibrillation	22(3.6%)	12(3.5%)	10(3.8%)	0.98
Long Hospital Stay(>10 Days)	301(49.3%)	97(28.6%)	204(75.0%)	<0.0001
1 Year Readmission(186 Subjects)	10 in 186(5.4%)	2 in 119(1.7%)	8 in 67(11.9%)	0.008
30- Day Mortality	11(1.8%)	2(0.6%)	9(3.3%)	0.03

FBS = Fasting Blood Sugar, TC = Total Cholesterol, LDL = Low Density Lipoprotein, HDL = High Density Lipoprotein, TG = Triglyceride, PP = Pulse Pressure, MAP = Mean Arterial Pressure, GFR = Glomerular Filtration Rate, 30-day Mortality = Death within 30 days of admission due to complications of heart failure.

### Echocardiography Profile of the Subjects

Table 2 shows the echocardiography profile of the subjects. There was no difference in the LV wall thickness or LV mass indexed for height ^2.7^ between subjects with reduced RV systolic function and those with normal RV systolic function. Subjects with reduced RV systolic function, however had larger left and right atrial and ventricular dimensions, lower LV ejection fraction and were more likely to have tricuspid regurgitation (TR) compared to the subjects with normal RV systolic function.

**Table 2:** Echocardiographic Profile of Subjects.

Parameters	ALL(611)	TAPSE≥15mm(339)	TAPSE<15mm(272)	P-value
RVD, cm	3.4±0.5	3.3±0.6	3.6±0.5	<0.0001
Left Atrium, cm	4.4±0.9	4.3±0.9	4.7±0.8	<0.0001
IVSD, cm	0.97±0.3	0.96±0.2	0.99±0.3	0.16
PWD, cm	1.1±0.2	1.08±0.2	1.12±0.2	0.08
EDD, cm	5.8±1.1	5.7±1.1	5.9±1.1	0.04
ESD, cm	4.7±1.3	4.6±1.2	4.9±1.3	0.003
LAA, cm^2^	24.5±7.0	23.3±6.9	26.8±8.4	<0.0001
RAA, cm^2^	20.3±8.1	18.7±7.7	23.9±8.4	<0.0001
LVM/ HT^2.7^	108.3±46.3	108.7±39.1	112.5±42.3	0.65
LVEF, %	35.2±17.5	40.7±16.8	32.3±7.9	<0.0001
ME, metres/second	0.78±0.3	0.79±0.3	0.83±0.3	0.22
MA, metres/second	0.56±0.2	0.53±0.2	0.64±0.1	0.61
ME/MA	2.1±1.3	1.9±1.2	2.3±1.4	<0.0001
DT, Milliseconds	143.2±80.6	147.9±77.8	125.3±72.2	0.22
Tricuspid Regurgitation	117(29.0%)	69(20.4%)	108(39.7%)	0.003
TAPSE, mm	16.4±4.9	19.9±3.6	12.1±1.9	<0.0001
TAPSE< 15mm	272(42.9%)	0(0%)	272(100%)	0.18
RVSP, mmHg	43.5±10.5	38.0±5.5	47.5±8.7	0.04

RVD = Right Ventricular Diameter in Diastole, IVSD = Inter-ventricular septal diameter in diastole, PWD = Posterior Wall Diameter in Diastole, EDD = End Diastolic Diameter, ESD = End Systolic Diameter, LVM = Left Ventricular Mass, ME = Early Mitral Inflow, MA = Late Mitral Inflow, LV EF = Left Ventricular Ejection Fraction, TAPSE = Tricuspid Annular Plane Systolic Excursion, DT = Deceleration time.

### Relationship between RV Function and Echocardiography and Laboratory Parameters

Table 3 shows the relationship between RV systolic function as assessed by TAPSE and various echocardiography and laboratory parameters. Left atrial diameter, left and right atrial area, LV end-diastolic and end-systolic diameters, LV mass index, RV systolic pressure, LV ejection fraction and transmitral E/A ratio correlated significantly with TAPSE.

**Table 3:** Relationship between RV Function and Echocardiography and Laboratory Parameters.

Parameters	Pearson Correlation	P-value
Left Atrial Diameter	-0.54	<0.0001
Total Cholesterol	-0.025	0.79
Body Mass Index	-0.30	0.82
EDD	-0.47	<0.0001
ESD	-0.55	<0.0001
LVM/HT2.7	-0.34	<0.0001
LVEF	0.60	<0.0001
Mitral E/A	-0.39	<0.0001
Left Atrial Area	-0.24	0.02
Right Atrial Area	-0.44	<0.0001
RVSP	-0.74	<0.0001
RVD	-0.035	0.77

EDD = End Diastolic Diameter, ESD = End Systolic Diameter, LVM = Left Ventricular Mass, ME = Early Mitral Inflow, MA = Late Mitral Inflow, LVEF = Left Ventricular Ejection Fraction, RVD = Right Ventricular Diameter, RVSP = Right Ventricular Systolic Pressure.

### Independent Predictors of RV Systolic Dysfunction

Table 4 shows that the Independent predictors of RV systolic dysfunction are poor LV function and increased right atrial area.

**Table 4:** Independent Co-variants of Right Ventricular Systolic Dysfunction using TAPSE.

Parameters	Pearson Correlation	P-value
LVEF	0.15	0.008
FS	0.30	0.006
RAA	0.44	<0.0001
Long hospital stay>10days	0.26	0.005

LVEF = Left Ventricular Systolic Function, FS = Fractional Shortening, RAA = Right Atrial Area.

## Discussion

Our study has shown the influence of hypertensive HF on RV function. Although hypertensive HF has been previously identified as the commonest cause of left-sided HF in sub-Saharan Africa [17–19], we are not aware that the effect on RV function has been previously reported on a sample size as large as 611. And even though right ventricular function has been studied in Nigerian hypertensive subjects, these studies were in those without heart failure and in a smaller cohort [20, 21].

We found RV systolic dysfunction in 272 (44.5%) of the 611 subjects studied. Although coronary artery disease is a recognized confounder for the development of RV systolic dysfunction in the setting of hypertension and HF, we have previously shown that coronary artery disease is relatively uncommon in HF patients in urban Nigeria(7), and is therefore unlikely to explain our findings.

The high prevalence of RV systolic dysfunction in our hypertensive HF cohort might partly explain the poor prognosis that is found in our subjects[19], as RV systolic dysfunction is an established adverse prognostic marker in HF[16,22]. This is further supported in our study as subjects with right ventricular systolic dysfunction (TAPSE<15mm) had worse prognosis compared to those with TAPSE≥15mm as shown by longer hospital stay, higher intra hospital mortality and higher 1-year re- hospitalization. Thus there is a need to pay more attention to RV function, which has previously been neglected in hypertensive HF subjects. The impairment of RV function in left-sided HF has been linked to ventricular interdependence [23]., which is often present in HF, being most apparent with changes in loading conditions such as those seen with volume loading[24,25].

29% of our subjects had tricuspid (TR), and expectedly, TR was more common in the subjects with RV systolic dysfunction. The mechanism of TR in our subjects was largely functional and has been linked to RV dilatation [26], rather than structural abnormalities of the tricuspid valvular leaflets. The prevalence of TR of 29% in our subjects is much higher than that in another African study, the Heart of Soweto Study [26], which may reflect the later presentation of our subjects when compared to those in Soweto.

On linear regression analysis, factors that were associated with abnormal RV function included dilated left atrium and LV, increased LV mass, reduced LV systolic function, restrictive filling pattern, and increased RV systolic pressure, while on multiple regression only low fractional shortening and LV ejection fraction, and increased right atrial area were determinants of right systolic dysfunction. LV dilation has been previously shown to have an impact on RV function as measured by TAPSE [21]. A reduced LV ejection fraction impacts on TAPSE even in the setting of preserved RV ejection fraction [23,27]. The effect of LV function on TAPSE has also been attributed to ventricular interdependence [16]. The finding in our study of an inverse relationship between RV systolic function and RVSP is in accordance with most previous studies [23]. This relationship has been reported in patients with dilated cardiomyopathy and ischemic heart disease, and suggests that the major determinant of RV systolic function in left-sided HF is after load mismatch, due to increase pulmonary artery resistance [22].

TAPSE correlated with markers of diastolic dysfunction and RV systolic pressure in our study, but not with RV end-diastolic diameter as previously reported [25]. This may suggest that the reduction in RV systolic function is not entirely explained by changes in filling pressure or hemodynamics. LV systolic dysfunction and right chamber dilatation as independent predictors of RV systolic dysfunction have been previously reported ([28, 29].

At presentation, most of our subjects had chamber dilatation especially of the left-sided chambers, and with significant systolic and diastolic dysfunction with an average LV ejection fraction (LVEF) of 35% and trans mitral E/A ratio of 2.1, suggesting raised LV filling pressure. The reduced LV systolic function in our hypertensive heart failure subjects is not surprising as systolic dysfunction has been linked to hypertension in a black population [30]. Subjects with impaired RV systolic function had significantly greater chamber dilatation in keeping with a previous study, which showed dilated cardiac chambers to be associated with RV systolic dysfunction[31]. In keeping with previous findings [32] subjects with RV systolic dysfunction had worse renal function and were more likely to present with palpitations and peripheral edema.

The diagnosis of ischaemic heart disease was made clinically from history, physical examination, troponin I levels and electrocardiography with no myocardial perfusion imaging or coronary angiography, and therefore there is the possibility that subclinical coronary artery disease may have been missed similar to our previous study [33]. However, this confounding factor must have been minimized with the careful and extensive phenotyping of our study population with respect to clinical, electrocardiographic, echocardiography and biochemical parameters.

## Conclusions

Hypertensive heart failure is a major cause of RV systolic dysfunction in Nigeria, which was present in over 40% of such patients. Importantly, this occurred despite a low prevalence of clinical ischemic heart disease in our cohort. In addition, RV systolic dysfunction is also associated with poorer prognosis in hypertensive HF. Detailed RV assessment should therefore be part of the echocardiography assessment of hypertensive HF.
